# Exploring quality of life in Australian adults during a pandemic

**DOI:** 10.1007/s12144-022-03831-3

**Published:** 2022-11-10

**Authors:** Caitlin Liddelow, Courtney S. Hitchcock, Barbara A Mullan

**Affiliations:** 1 Global Alliance for Mental Health and Sport, School of Psychology, University of Wollongong, 2500 AUS Wollongong, NSW Australia; 2 EnAble Institute, Curtin University, 6102 AUS Perth, WA Australia

**Keywords:** COVID-19 anxiety, Adaptive coping, Health risk, Lessons learnt, Reflections

## Abstract

Many aspects of society changed due to the COVID-19 pandemic. As a result, many individuals experienced the introduction of travel bans and restrictions, COVID-19 related anxiety, greater risk to their health and an increased need for adaptive coping. Research has shown health-related quality of life was negatively affected during the time. However, the influence that these restrictions and experiences had on other various quality of life domains (physical, psychological, environmental, and social) is not yet known. Therefore, we aimed to examine the relationships between COVID-19-related variables, health variables, psychological variables and five domains of quality of life in Australian adults. Data was collected via cross-sectional online surveys from 264 Australian participants (*M*_*age*_ = 29.76 years, *SD* = 12.40). Five hierarchical multiple regression analyses were conducted. The findings showed better adaptive coping, decreased COVID-19 anxiety, and lower perceived health risk were all associated with better quality of life during this time. Neither having travel plans during 2020–2021 nor engaging in compensatory behaviours were associated with quality of life. During times of uncertainty, such as pandemics, natural disasters or war, providing anxiety-reducing coping strategies may be beneficial for reducing the negative impacts on quality of life. In line with these findings and similar research, we have provided several directions and recommendations for governments and media organisations for when future events, similar to COVID-19, occur.

Whilst the world may have grappled with COVID-19 for the last few years, pandemics and epidemics are not unusual. The world and its people have struggled through similar occurrences before, such as the plague, Chorea and even influenza (Piret & Boivin, 2021). However, much of the world’s current population had not experienced a pandemic like COVID-19, therefore bringing many of the health and social issues associated with pandemics and times of uncertainty, to the forefront. From this experience with COVID-19 (Shek, 2021), society must learn to efficiently adapt and cope with changes during times of uncertainty. We, as scientists, psychologists, and researchers, need to provide directions and recommendations based on theory for people in times of uncertainty to ensure quality of life is maintained. It is likely the world will experience similar situations again, whether it is another pandemic, a war or travel restrictions due to other events.

In December 2019, COVID-19 spread rapidly throughout the world, resulting in a global pandemic that is still ongoing. This pandemic interfered with everyday life, including closures of schools and public places, changes to work and home routines, and the introduction of many hygiene measures (Hagedorn et al., 2022; Vieira & Meirinhos, 2021). Combined, the restrictions placed on many aspects of life, and the uncertainty that many felt may have impacted quality of life. The long-term negative psychological and physical effects that restrictions have had has been well emphasised (Kowal et al., 2020; Marques et al., 2020; Peteet, 2020). For example, research in the initial month of the pandemic showed many Australian adults displayed clinically significant symptoms of anxiety and depression during the imposed lockdown (Fisher et al., 2020), directly affecting quality of life. More recent research has shown that parents and caregivers in Australia who were required to home-school children during the pandemic experienced higher levels of psychological distress, which also negatively impaired their work and social life, compared to those who were not required to home-school (Calear et al., 2022).

One important COVID-19-related restriction that has received less focus with regards to its effect on wellbeing and quality of life are the travel restrictions that were imposed in many countries globally. The pandemic rapidly changed travel on an unparalleled scale, with travel within and between countries restricted (Ioannides & Gyimóthy, 2020; Osofsky et al., 2020). In December 2019, prior to the initial COVID-19 outbreak, there were 2.24 million international departures of Australian residents (Australian Bureau of Statistics, 2021). This figure does not include domestic flights, cruise ships, or other forms of transportation. By March 2020, this figure dropped to 830,000 international departures, with a further drop to 30,000 departures by May 2020 (Australian Bureau of Statistics, 2021), indicating that many had their travel plans cancelled, postponed, or changed in response to travel restrictions.

People travel for many different reasons, such as for work, cultural emersion, or volunteering (Lew, 2018), and a positive relationship exists between leisure travel and wellbeing (Coghlan, 2015). Leisure travel can lead to an increase in consciousness and identity development, and a greater sense of creativity, connectedness, and acceptance of diversity (Lew, 2018), which all reflect positively on wellbeing. Whilst research has shown that travel restrictions during the pandemic were successful in reducing the spread of COVID-19 (Bou-Karroum et al., 2021; Kwok et al., 2021), it is not known how these restrictions may have influenced the quality of life of many individuals. With the inability to travel, many individuals may not have had the opportunity to experience the positive benefits that are associated with leisure travel (Coghlan, 2015). It is therefore important to consider the effect of these travel restrictions, in conjunction with other COVID-19 related factors, on the quality of life of individuals.

Further, other proximal factors may have also impaired life quality during this time of uncertainty, such as an individual’s anxiety related to the pandemic (Brosschot et al., 2016; Özdin & Bayrak Özdin, 2020), their perceived health risk of COVID-19 infection (Chua et al., 2021), and their current health status (Dennison et al., 2022). For example, a study of Greek adults with chronic disease showed they reported significantly higher levels of distress during the COVID-19 pandemic compared to individuals without a chronic disease (Louvardi et al., 2020). Furthermore, individuals with respiratory diseases reported the highest levels of distress (Louvardi et al., 2020), suggesting those who perceived themselves as potentially more susceptible to COVID-19 experienced greater distress. Similarly, individuals with greater health impairment often report lower levels of quality of life during typical times (Fischer et al., 2009), however, recent research has suggested quality of life for individuals with health impairments worsened during COVID-19 (Dennison et al., 2022).

Until mid-2021, many Australians had not experienced the first-hand exposure or effects of COVID-19, and therefore it may have been difficult for members of the broader Australian population to understand the severity of it. Due to this, Australians tended to experience COVID-19 via news broadcasting channels and secondary sources. As such, there was an escalation in anxiety levels about the uncertainty of COVID-19 in many Australians (Faasse & Newby, 2020), which led to many unique COVID-19-related anxiety symptoms (Silva et al., 2020). Therefore, many individuals had to develop creative coping techniques, such as engaging in new behaviours, to preserve their mental health and quality of life.

## Stress and Coping During Times of Uncertainty

Fredrickson’s broaden-and-build theory of positive emotions (Fredrickson, 2001) suggests that when individuals can freely explore and take in new information and experiences from the world around them, they can develop their sense of self, feel positive emotional interest and develop greater social, psychological, and intellectual resources. The experience of negative emotions reduces the ability to cope with stressful or uncertain situations. Considering this theory in the context of COVID-19, such as restrictions to travel and not being able to experience the positive benefits associated with travel (Coghlan, 2015), or perceived heightened risk to health, and therefore experiencing greater psychological distress (Louvardi et al., 2020), it is likely this would have diminished the quality of life of many.

Furthermore, Lazarus and Folkman (1984) suggest that coping throughout a stressful encounter is determined in part by an individual’s resources, including their health, beliefs about control, social support, and problem-solving skills. During the COVID-19 pandemic, many experienced the decline of in-person social connection, autonomy to travel, and ease of health-related help-seeking (both physical and psychological). These coping resources that typically were readily available in times of difficulty were reduced or removed entirely (Auerbach & Miller, 2020). Thus, individuals may have adaptively coped by engaging in new compensatory behaviours, such as volunteering (Same et al., 2020) or yoga (Sahni et al., 2021) to ‘fill the void’ that some COVID-19 related restrictions created. Sahni et al. (2021) found that engaging in yoga was an effective self-management strategy to cope with stress, anxiety, and depression that stemmed from the pandemic. Similarly, due to the closure of fitness facilities and the inability to attend live sporting events during the pandemic, sport consumers reported higher impulse buying of sports good as a way to cope (Cho et al., 2021). Therefore, it would be expected that individuals who had greater levels of adaptive coping and engaged in new compensatory behaviours would have better quality of life during this time.

## The Current Research

This exploratory study aims to understand how COVID-19 related travel variables (e.g., previous travel plans), health-related variables (e.g., health impairment, health risk perception), and psychological variables (e.g., COVID-19 anxiety, adaptive coping, compensatory behaviours) have influenced Australian adults’ quality of life during a global pandemic. It is hypothesised that:

### H1

Informed by Fredrickson’s (2001) broaden-and-build theory, having plans to travel over the past year (mid 2020-mid 2021) will be associated with poorer quality of life during this time.

### H2

In-line with previous research, greater health impairment and higher perceived health risk from COVID-19 will both be associated with poorer quality of life.

### H3

Based on previous research, higher COVID-19 anxiety will be associated with poorer quality of life.

### H4

In-line with Lazarus and Folkman (1984), adaptive coping will be positively associated with quality-of-life outcomes, and.

### H5

Engaging in behaviours or activities to compensate for lack of travel will be associated with better quality of life.

## Method

### Measures

### Quality of life

Quality of life was measured using the World Health Organization Quality of Life scale, brief version (WHOQOL -BREF). There are two broad questions assessing individuals’ perceptions of their overall life quality and health, followed by 24 questions assessing physical, psychological, social, and environmental domains (THE WHOQOL GROUP, 1998). Higher scores represent perceptions of better quality of life in the given life areas. Cronbach’s alpha for the entire scale in this sample was α = 0.90, which is consistent with previous research.

### Travel Behaviour

Travel behaviour was measured using seven items that were created for this research. The questions related to participants’ typical travel behaviour in a given year, any plans they had during the past year to travel, how those plans were impacted by the COVID-19 pandemic, as well any quarantining. Follow-up questions were asked if participants indicated that they had plans to travel (e.g., where, when, why). However, only the two items related to travel plans (domestic and international) in the previous year were included in the final analyses. Participants provided responses as either 1 (*Yes, I had plans to travel*) or 2 (*No, I did not have plans to travel*) for each of these two items.

### Compensatory behaviours

Participants with travel plans were asked if they had engaged in any behaviours or activities to compensate for their lack of travel. Participants were prompted to respond to the question either as 1 (*Yes*) or 2 (*No*).

### Perceived Health risk for COVID-19

Perceived health risk to contracting COVID-19 was measured by asking *“How do you view your health in relation to COVID-19?”* with possible answers ranging from, 1 (*I don’t know or would rather not say what I think about COVID-19 in relation to my health*), to 5 (*I feel COVID-19 poses a large threat to my health*). Higher scores indicated a greater risk perception of COVID-19 concerning their health.

### Health Impairment

To measure health impairment, one item asking participants to “*Please select any of the following health conditions that apply to you and significantly impact your everyday life”.* The 12 conditions listed were derived from the People with Disability in Australia report from the Australian Institute of Health and Welfare (2020). Conditions included, but were not limited to, mental and neurological disorders, muscoskeletal disorders, chronic respiratory diseases, and cardiovascular diseases. The more health conditions selected, the greater the health impairment.

### COVID-19 anxiety

The COVID-19 Anxiety Scale contains seven items asking participants to select an answer that best described how they felt over the past year (Silva et al., 2020). Items were scored on a four-point Likert-type scale from 0 (*not at all*) to 3 (*nearly every day*). For example, “*I feel uneasy when reading news about COVID-19”*. Scores were averaged to produce an overall anxiety score and ranged from 1 to 4, where higher scores indicated higher anxiety. Cronbach’s alpha for this sample was α = 0.87.

### Adaptive coping

Adaptive coping was measured by the Brief Resilience Coping Scale, which contains four items assessing an individual’s tendency to cope with challenging circumstances (Sinclair & Wallston, 2004). Items were scored on a five-point Likert-type scale from 1 (*Does not describe me at all*) to 5 (*Describes me very well)*. For example, “*I look for creative ways to alter difficult situations”*. Scores were summed to produce an overall score ranging from 4 to 20. Higher scores suggested a strong belief in their ability to adaptively deal with adverse life events. Cronbach’s alpha was α = 0.69.

## Procedure

### Ethics approval

from received from the University’s Human Research Ethics Committee (HRE2021-0143) prior to data collection. Data was collected via Qualtrics between June and September 2021. An *a*-priori power analysis was conducted using G*power with an estimated effect size of *f²* = 0.065, power of = 0.80, error = 0.05, and 11 predictors, which indicated that a sample of 269 was necessary. Participants were recruited via social media (e.g. Facebook and Twitter), flyers around the University campus and via the University student participant pool. All participants were required to be at least 16 years of age, be an Australian resident, understand written English, and have access to the internet.

The questionnaire was anonymous, and participation was voluntary. Informed consent was also obtained before participation. If participants were aged between 16 and 18 years, they were required to answer additional questions to demonstrate that true informed consent was obtained. Two questions and a CAPTCHA were included after the consent question to ensure responses from bots were not included. The survey was block randomised and took approximately 15 minutes to complete. Participants who were recruited through the student participant pool received one point for their participation.

### Data Analysis

The data associated with this study can be accessed on the Open Science Framework (https://osf.io/yfsqp/?view_only=c584bbb164f64a7385a7a434aa595355). The raw data was transferred from Qualtrics to IBM SPSS (Version 27) where it was cleaned and screened. Missing values analysis was completed before cases were deleted, and the data was deemed to be missing completely at random as per Little’s MCAR test, and not systematically connected to other variables *χ*² (626) = 678.05, *p* = .073. After case deletion, expectation maximisation replaced the remaining missing data points.

Appropriate assumptions were tested, and bivariate correlations were calculated before running the final regression analyses. Bivariate correlations showed that gender was the only demographic variable significantly correlated with quality-of-life measures, and thus was the only demographic variable controlled for in analyses. Age and education level were not significantly associated with any of the outcomes. Bivariate correlations and descriptive statistics for variables included in the final analyses are displayed in Table 1.

**Table 1:** *Descriptive Statistics and Bivariate Correlations Among the Variables of Interest*

	*M*	*SD*	2	3	4	5	6	7	8	9	10	11	12	13	14	15
**1. Gender**	-	-	− 0.07	− 0.08	0.29**	0.03	− 0.09	− 0.10	− 0.04	− 0.19**	0.04	− 0.08	− 0.13*	− 0.13*	− 0.09	− 0.16**
**2. Age (years)**	29.76	12.40	-	0.03	0.13*	0.10	− 0.12	0.08	− 0.24**	− 0.11	− 0.06	0.00	− 0.04	0.03	− 0.09	0.04
**3. Education**	-	-		-	− 0.01	− 0.02	0.02	0.16*	0.04	− 0.02	0.01	0.08	0.11	0.06	0.02	0.08
**4. Health impairment**	0.50	1.01			-	0.03	− 0.06	− 0.01	0.02	0.14	− 0.08	− 0.10	− 0.37**	− 0.25**	− 0.09	− 0.17**
**5. Health risk - COVID-19**	3.02	1.46				-	− 0.05	0.11	0.01	0.23**	0.20	− 0.05	− 0.17*	− 0.05	− 0.07*	− 0.11
**6. International travel plans**	-	-					-	− 0.03	0.09	0.08	− 0.01	− 0.05	0.02	0.01	0.00	0.03
**7. Domestic travel plans**	-	-						-	− 0.04	0.10	0.00	0.07	0.02	0.04	0.07	0.10
**8. Compensatory behaviours**	-	-							-	0.09	0.20**	0.15*	0.05	0.04	0.01	− 0.05
**9. COVID-19 Anxiety**	2.46	0.69								-	− 0.12*	− 0.12*	− 0.25**	− 0.23**	− 0.03	− 0.27**
**10. Adaptive Coping**	15.32	2.78									-	0.31**	0.26**	0.44**	0.21**	0.34**
**11. Overall Quality of life**	4.19	0.74										-	0.50**	0.48**	0.23**	0.54**
**12. Physical Quality of life**	110.50	17.45											-	0.67**	0.33**	0.52**
**13. Psychological Quality of life**	80.53	16.01												-	0.44**	0.55**
**14. Social Quality of life**	44.00	11.01													-	0.36**
**15. Environmental Quality of life**	126.80	17.25														-

*Note.* *p < .05, **p < .01. Gender (1 = female, 2 = male). Education (1 = less than a bachelor’s degree, 2 = bachelor’s degree or higher). International/Domestic travel plans (0 = no plans during the past year, 1 = plans to travel during the past year). Compensatory behaviours (0 = did not engage, 1 = did engage)

Five hierarchical multiple regression analyses were conducted. The criterion variable for the first regression was overall quality of life. The following regression models each had physical, psychological, social, and environmental quality of life as criterion variables. For all analyses, gender was controlled in block one. Health impairment and perceived health risk from COVID-19 were entered in block two. Travel plans (international and domestic) were entered in block three. The psychological variables measuring COVID-19-related anxiety, adaptive coping, and engagement in compensatory behaviours were added to block four.

## Results

### Sample

A total of 366 participants accessed the survey. Of whom, 102 cases were deleted due to missing data (over 30% total data or 20% of criterion data), failing to pass bot checks, or for not meeting inclusion or consent criteria. After data cleaning, a sample of 264 remained. Participants were aged between 18 and 76 years (*M* = 29.76, *SD* = 12.40), with 80.68% identifying as female (*n* = 213), 17.80% as male (*n* = 47), and 1.51% as non-binary (*n* = 4). The majority of participants were from Western Australia (WA; 83.70%). All participants were employed or were receiving government assistance, and slightly over half (58.70%) had participated in some form of higher education (e.g., bachelor’s degree).

## Overall quality of life

In block one, gender accounted for a non-significant 0.8% (*p* = .155) of the variance in overall quality of life. In block two, health impairment and perceived health risk from COVID-19 accounted for an additional non-significant 0.9% (*p* = .234) of variance. In block three, participants’ plans to travel accounted for an additional non-significant 0.9% (*p* = .302) of the variance. In block four, COVID-19 anxiety, adaptive coping and engagement in compensatory behaviours accounted for a statistically significant 12.3% (*p* < .001) of the variance. The overall model accounted for a statistically significant 15% of variance in overall quality of life scores, *R*^*2*^ = 0.15, *F* (8, 252) = 5.52, *p* < .001. Adaptive coping was the only significant predictor in the final model. This result had a moderate effect size, *f*^*2*^ = 0.18 (Cohen, 1988). Table 2 shows the coefficients and model summaries for this regression.

**Table 2:** *Coefficients of Hierarchical Multiple Regression Predicting Overall Quality of Life*

	Variable	*B* [95% CI]	$\varvec{\beta }$	*sr* ^2^	*p-*value	*R* ^2^	Δ*R*^2^	*F*	Δ*F* [*df*1, *df*2]
**Step 1**				-	0.155	0.01	-	2.03	2.03 [1, 259]
	Gender	− 0.14 [-0.33, 0.05]	− 0.09	0.00	0.155				
**Step 2**				-	0.234	0.02	0.01	1.43	1.13 [2, 257]
	Gender	− 0.10 [-0.31, 0.10]	− 0.07	0.00	0.314				
	Health Impairment	− 0.05 [-0.15, 0.004]	− 0.07	0.00	0.251				
	Health risk – COVID-19	− 0.03 [-0.09, 0.03]	− 0.06	0.00	0.351				
**Step 3**				-	0.247	0.03	0.01	1.34	1.20 [2, 255]
	Gender	− 0.10 [-0.30, 0.011]	− 0.06	0.00	0.342				
	Health Impairment	− 0.06 [-0.15, 0.04]	− 0.08	0.01	0.233				
	Health risk – COVID-19	− 0.04 [-0.10, 0.03]	− 0.07	0.00	0.269				
	International travel plans	− 0.09 [-0.30, 0.11]	− 0.06	0.00	0.361				
	Domestic travel plans	0.12 [-0.08, 0.32]	0.08	0.01	0.224				
**Step 4**					< 0.001**	**0.15**	**0.10**	**5.60**	**14.75 [3, 252]**
	Gender	− 0.17 [-0.37, 0.03]	− 0.11	0.01	0.090				
	Health Impairment	− 0.02 [-0.11, 0.07]	− 0.03	0.00	0.665				
	Health risk – COVID-19	− 0.03 [-0.09, 0.03]	− 0.05	0.00	0.397				
	International travel plans	− 0.09 [-0.28, 0.10]	− 0.05	0.00	0.370				
	Domestic travel plans	0.14 [-0.05, 0.33]	0.09	0.01	0.144				
	COVID-19 anxiety	− 0.13 [-0.26, 0.01]	− 0.12	0.01	0.064				
	Adaptive coping	0.08 [0.05, 0.11]	0.29	0.08	< 0.001**				
	Compensatory Behaviours	0.16 [-0.02, 0.33]	0.11	0.01	0.081				

*Note:* CI – confidence interval. *p < .05. **p < .01. Significant steps are bolded.

### Physical quality of life

In block one, gender accounted for a statistically significant 1.9% (*p* = .026) of the variance. In block two, health impairment and perceived health risk for COVID-19 accounted for an additional significant 15.2% (*p* < .001) of the variance. In block three, travel plans in the previous year accounted for an additional non-significant 0.2% (*p* = .786) of the variance. In block four, COVID-19 anxiety, adaptive coping and engagement in compensatory behaviours added a statistically significant 8.7% (*p* < .001) of the variance. The overall model explained 25.9% of the variance in physical quality of life, *R*^*2*^ = 0.26, *F* (8, 252) = 11.01, *p* < .001. Health impairment, health risk for COVID-19, COVID-19 anxiety, and adaptive coping were significant in the final model. These results had a medium to large effect (*f*^*2*^ = 0.35) according to Cohen (1988). Table 3 shows the coefficients and model summaries for this regression.

**Table 3:** *Coefficients of Hierarchical Multiple Regression Predicting Physical Quality of Life*

	Variable	*B* [95% CI]	$\varvec{\beta }$	*sr* ^2^	*p* value	*R* ^2^	Δ*R*^2^	*F*	Δ*F* [*df*1, *df*2]
**Step 1**				-	0.026*	**0.02**	**-**	**4.99**	**4.99 [1, 259]**
	Gender	-5.19 [-9.76, -0.61]	− 0.14	0.02	0.026*				
**Step 2**				**-**	< 0.001**	**0.17**	**0.15**	**17.61**	**23.49 [2, 257]**
	Gender	-1.03 [-5.45, 3.38]	− 0.03	0.00	0.646				
	Health Impairment	-6.26 [-8.30, -4.25]	− 0.36	0.12	< 0.001**				
	Health risk - COVID-19	-1.95 [-3.29, -0.62]	− 0.16	0.03	0.004**				
**Step 3**				**-**	< 0.001**	**0.17**	**0.00**	**10.60**	**0.24 [2, 255]**
	Gender	− 0.91 [-5.37, 3.55]	− 0.02	0.00	0.688				
	Health Impairment	-6.27 [-8.29, -4.25]	− 0.37	0.12	< 0.001**				
	Health risk - COVID-19	-2.01 [-3.36, − 0.067]	− 0.17	0.03	0.004**				
	International travel plans	− 0.34 [-4.43, 4.05]	− 0.01	0.00	0.878				
	Domestic travel plans	1.49 [-3.88, 5.85]	0.04	0.00	0.503				
**Step 4**				**-**	< 0.001**	**0.26**	**0.09**	**11.01**	**9.86 [3, 252]**
	Gender	-3.18 [-7.57, 1.21]	− 0.08	0.01	0.155				
	Health Impairment	-5.26 [-7.23, -3.29]	− 0.31	0.08	< 0.001**				
	Health risk - COVID-19	-1.60 [-2.93, -0.29]	− 0.14	0.02	0.017*				
	International travel plans	0.27 [-3.94, 4.47]	0.01	0.00	0.901				
	Domestic travel plans	1.86 [-2.30, 6.03]	0.05	0.00	0.379				
	COVID-19 anxiety	-4.49 [-7.44, -1.54]	− 0.18	0.03	0.003*				
	Adaptive coping	1.41 [0.72, 2.10]	0.23	0.05	< 0.001**				
	Compensatory behaviours	0.71 [-3.22, 4.64]	0.02	0.00	0.724				

*Note:* CI – confidence interval. *p < .05. **p < .01. Significant steps are bolded.

### Psychological quality of life

In block one, gender accounted for a statistically significant 2.0% (*p* = .022) of the variance in psychological quality of life. In block two, health impairment, and perceived health risk for COVID-19 accounted for an additional significant 4.9% (*p* < .001) of the variance. In block three, travel plans accounted for a non-significant additional 0.1% (*p* = .820) of the variance. In the final block, COVID-19 anxiety, adaptive coping, and compensatory behaviours explained a significant additional 22.8% (*p* < .001) of the variance. The total model accounted for 29.8% of the variance, *R*^*2*^ = 0.30, *F* (8, 252) = 13.38, *p* < .001, with a large effect size of *f*^*2*^ = 0.43 (Cohen, 1988). The only significant predictors in the final model were gender, health impairment, COVID-19 anxiety, and adaptive coping. See Table 4 for the coefficients and model summaries for this regression.

**Table 4:** *Coefficients of Hierarchical Multiple Regression Predicting Psychological Quality-of-Life*

	Variable	*B* [95% CI]	$\varvec{\beta }$	*sr* ^2^	*p* value	*R* ^2^	Δ*R*^2^	*F*	Δ*F* [*df*1, *df*2]
**Step 1**				-	0.022*	**0.02**	**-**	**5.28**	**5.28 [1, 259]**
	Gender	-4.91 [-9.12, -0.70]	− 0.14	0.02	0.022*				
**Step 2**				**-**	< 0.001**	**0.07**	**0.05**	**6.35**	**6.77 [2, 257]**
	Gender	-2.59 [-6.90, 1.71]	− 0.08	0.01	0.237				
	Health Impairment	-3.55 [-5.51, 1.59]	− 0.22	0.05	< 0.001**				
	Health risk - COVID-19	− 0.53 [-1.83, 0.77]	− 0.05	0.00	0.421				
**Step 3**				**-**	0.002**	**0.07**	**0.00**	**3.87**	**0.20 [2, 255]**
	Gender	-2.47 [-6.82, 1.89]	− 0.07	0.00	0.266				
	Health Impairment	-3.56 [-5.53, 1.59]	− 0.23	0.05	< 0.001**				
	Health risk - COVID-19	− 0.58 [-1.90, 0.73]	− 0.05	0.00	0.384				
	International travel plans	− 0.10 [-4.39, 4.18]	0.00	0.00	0.962				
	Domestic travel plans	1.35 [-2.90, 5.61]	0.04	0.00	0.532				
**Step 4**				**-**	< 0.001**	**0.30**	**0.23**	**13.38**	**27.24 [3, 252]**
	Gender	-5.35 [-9.29, -1.42]	− 0.15	0.02	0.008**				
	Health Impairment	-2.20 [-3.97, -0.44]	− 0.14	0.02	0.015*				
	Health risk - COVID-19	− 0.20 [-1.39, 1.00]	− 0.02	0.00	0.736				
	International travel plans	0.77 [-3.01, 4.54]	0.02	0.00	0.689				
	Domestic travel plans	1.62 [-2.11, 5.35]	0.05	0.00	0.393				
	COVID-19 anxiety	-4.58 [-7.22, -1.94]	− 0.20	0.03	< 0.001**				
	Adaptive coping	2.47 [1.85, 3.09]	0.43	0.17	< 0.001**				
	Compensatory behaviours	-1.38 [-4.90, 2.14]	− 0.04	0.00	0.440				

*Note:* CI – confidence interval. *p < .05. **p < .01. Significant steps are bolded.

### Social quality of life

In one block, gender accounted for a statistically non-significant 0.8% (*p* = .147) of the variance. In block two, health impairment and perceived health risk from COVID-19 accounted for an additional non-significant 0.9% (*p* = .323) of variance. In block three, travel plans accounted for an additional non-significant 0.4% (*p* = .592) of the variance. In block four, COVID-19 anxiety, adaptive coping and compensatory behaviours accounted for an additional significant 4.5% (*p* = .008) of the variance. The overall model accounted for a significant 6.6% of variance, *R*^*2*^ = 0.07, *F* (8, 252) = 2.22, *p* = .026, but had a small to moderate effect size (*f*^*2*^ = 0.08). The only significant predictor in the final model was adaptive coping. See Table 5 for the coefficients and model summaries for this regression.

**Table 5:** *Coefficients of Hierarchical Multiple Regression Predicting Social Quality-of-Life*

	Variable	*B* [95% CI]	$\varvec{\beta }$	*sr* ^2^	*p* value	*R* ^2^	Δ*R*^2^	*F*	Δ*F* [*df*1, *df*2]
**Step 1**				-	0.147	0.01	-	2.11	2.11 [1, 259]
	Gender	-2.15 [-5.06, 0.76]	− 0.09	0.01	0.147				
**Step 2**				-	0.225	0.02	0.01	1.46	1.14 [2, 257]
	Gender	-1.64 [-4.68, 1.40]	− 0.07	0.00	0.288				
	Health Impairment	-0.73 [-2.12, 0.66]	− 0.07	0.00	0.300				
	Health risk - COVID-19	-0.50 [-1.41, 0.42]	− 0.06	0.00	0.289				
**Step 3**				-	0.369	0.02	0.00	1.08	0.52 [2, 255]
	Gender	-1.50 [-4.57, 1.57]	− 0.07	0.00	0.336				
	Health Impairment	-0.74 [-2.13, 0.65]	− 0.07	0.00	0.295				
	Health risk - COVID-19	-0.55 [-1.48, 0.37]	− 0.07	0.01	0.241				
	International travel plans	-0.17 [-3.19, 2.86]	− 0.01	0.00	0.914				
	Domestic travel plans	1.55 [-1.46, 4.55]	0.06	0.00	0.311				
**Step 4**				**-**	0.026*	**0.07**	**0.05**	**2.22**	**4.05 [3, 252]**
	Gender	-1.93 [-5.05, 1.19]	− 0.08	0.01	0.225				
	Health Impairment	-0.49 [-1.89, 0.91]	− 0.05	0.00	0.492				
	Health risk – COVID-19	-0.59 [-1.53, 0.35]	− 0.08	0.01	0.216				
	International travel plans	-0.05 [-3.04, 2.94]	− 0.00	0.00	0.975				
	Domestic travel plans	1.50 [-1.46, 4.46]	0.06	0.00	0.320				
	COVID-19 anxiety	-0.01 [-2.08, 2.10]	0.00	0.00	0.993				
	Adaptive coping	0.86 [0.37, 1.35]	0.22	0.04	< 0.001*				
	Compensatory behaviours	-0.75 [-3.55, 2.04]	− 0.03	0.00	0.596				

*Note:* CI – confidence interval. *p < .05. **p < .01. Significant steps are bolded.

### Environmental quality of life

In block one, gender accounted for a significant 2.3% (*p* = .015) of the variance. In block two, health impairment and perceived health in relation to COVID-19 accounted for an additional significant 2.7% (*p* = .028) of variance. In block three, travel plans accounted for an additional non-significant 1.1% (*p* = .232) of variance. In block four, COVID-19 anxiety, adaptive coping, and engagement in compensatory behaviours were entered, accounting for an additional significant 18.6% (*p* < .001) of variance. The final model accounted for a statistically significant 24.6% of the variance in environmental quality of life, *R*^*2*^ = 0.25, *F* (8, 252) = 10.29, *p* < .001. Gender, domestic travel plans, COVID-19 anxiety and adaptive coping were the significant variables in the final model. This result had a medium to large effect of *f*^*2*^ = 0.33 (Cohen, 1988). See Table 6 for the coefficients and model summaries for this regression.

**Table 6:** *Coefficients of Hierarchical Multiple Regression Predicting Environmental Quality-of-Life*

	Variable	*B* [95% CI]	$\varvec{\beta }$	*sr* ^2^	*p* value	*R* ^2^	Δ*R*^2^	*F*	Δ*F* [*df*1, *df*2]
**Step 1**				**-**	0.015*	**0.02**	**-**	**6.03**	**6.03 [1, 259]**
	Gender	-5.61 [-10.11, -1.11]	− 0.15	0.02	0.015*				
**Step 2**				-	0.004**	**0.05**	**0.03**	**4.46**	**3.62 [2, 257]**
	Gender	-4.09 [-8.75, 0.58]	− 0.11	0.01	0.086				
	Health Impairment	-2.25 [-4.38, -0.13]	− 0.13	0.02	0.038*				
	Health risk - COVID-19	-1.17 [-2.58, 0.24]	− 0.10	0.01	0.103				
**Step 3**				**-**	0.007**	**0.06**	**0.01**	**3.28**	**1.47 [2, 255]**
	Gender	-3.64 [-8.33, 1.05]	− 0.10	0.01	0.128				
	Health Impairment	-3.26 [-4.39, -0.14]	− 0.13	0.02	0.037*				
	Health risk - COVID-19	-1.30 [-2.72, 0.11]	− 0.11	0.01	0.071				
	International travel plans	0.65 [-3.97, 5.26]	0.02	0.00	0.782				
	Domestic travel plans	3.96 [-0.62, 8.55]	0.11	0.01	0.090				
**Step 4**				**-**	< 0.001**	**0.25**	**0.19**	**10.28**	**20.71 [3, 252]**
	Gender	-7.16 [-11.53, -2.79]	− 0.19	0.03	0.001**				
	Health Impairment	-0.72 [2.68, 1.24]	− 0.04	0.00	0.468				
	Health risk - COVID-19	-0.70 [-2.01, 0.62]	− 0.06	0.00	0.298				
	International travel plans	1.93 [-2.26, 6.12]	0.05	0.00	0.365				
	Domestic travel plans	4.29 [0.15, 8.43]	0.11	0.01	0.042*				
	COVID-19 anxiety	-6.45 [-9.39, -3.52]	− 0.26	0.06	< 0.001**				
	Adaptive coping	2.08 [1.39, 2.77]	0.34	0.11	< 0.001**				
	Compensatory behaviours	-3.48 [-7.39, 0.44]	− 0.10	0.01	0.081				

*Note:* CI – confidence interval. *p < .05. **p < .01. Significant steps are bolded.

## Discussion

This research aimed to examine the relationships that exist between factors relating to COVID-19, travel restrictions, and quality of life outcomes in Australian adults. Our proposed exploratory model was partially supported. Between 7 and 30% of the variance in quality of life was accounted for by the variables measured. The model explained the most variance in environmental, and psychological quality of life. Adaptive coping was the most important predictor of quality of life during COVID-19, such that those with greater coping ability reported better quality of life across all five outcomes. This finding supports hypothesis five which proposed adaptive coping would be positively and significantly associated with quality of life. COVID-19 anxiety was also an important predictor of quality of life, being a significant predictor for three domains (physical, psychological and environmental). Individuals who reported greater levels of COVID-19 anxiety reported lower levels of quality of life, which supports hypothesis three. Partially supporting hypothesis two that having greater health impairment and a greater health risk for COVID-19 would be associated with poorer quality of life, health impairment and health risk for COVID-19 were significant predictors for physical quality of life, but no other quality of life domains. Contrary to hypotheses one and four, having travel plans during 2020–2021 or engaging in compensatory behaviours were not identified as being important predictors of quality of life.

Adaptive coping during the COVID-19-related travel restrictions in Australia appeared to be the only important predictor of overall quality of life. Individuals with greater coping skills reported greater quality of life overall, supporting recent research which identified the importance of adaptive coping strategies for ‘surviving’ the COVID-19 pandemic (Javed & Parveen, 2021; Meyer et al., 2022; Shamblaw et al., 2021), as well as for dealing with other times of uncertainty, such as a cancer diagnosis (Macía et al., 2020) or during political and military conflict (Hammad & Tribe, 2021). This finding also aligns with previous research which proposed COVID-19 has activated long-term stress responses in many people worldwide (Tintori et al., 2020) and therefore created a unique opportunity for many individuals to adapt to the uncertain conditions that continue to unfold and find nuanced ways of creating a sense of normalcy in everyday life (Tintori et al., 2020). The use of qualitative research methods may allow for future research to grasp more in-depth lived experiences of the pandemic and any adaptive coping skills that were used.

Related to adaptive coping, engagement in compensatory behaviours in the absence of travel was not a significant predictor of any quality-of-life outcomes. There are currently very few studies exploring the role of compensatory behaviours in the absence of travel during the pandemic. However, one explanation for this finding is that other coping mechanisms, beside engaging in compensatory behaviours, were employed during this time. For example, a recent study reported that many individuals used humour to adaptively cope with the COVID-19-related travel restrictions (Lenggogeni et al., 2022). This is further supported by our findings that those with greater levels of adaptive coping reported greater quality of life. Perhaps in times of uncertainty, compensatory behaviours are not as important for coping compared to other mechanisms.

In Australia, in the early months of the COVID-19 pandemic, approximately two-thirds of adults reported being worried about COVID-19 (Faasse & Newby, 2020). Our findings also showed that individuals who reported higher levels of COVID-19 anxiety reported poorer quality of life, specifically physical, psychological and environmental quality of life. This is not surprising and supports other global research in Canadian adults (Shoychet et al., 2022), Indian adults (Kharshiing et al., 2021), as well as healthcare workers in Iran (Mohamadzadeh Tabrizi et al., 2022). However, no studies have previously identified the relationship between COVID-19 anxiety and environmental quality of life. Environmental quality of life focuses on the aspects of life related to freedom, physical safety, work satisfaction, access and quality of health and social care, the physical environment, and opportunities and participation in recreational and leisure activities (THE WHOQOL GROUP, 1998). Individuals with higher levels of COVID-19 anxiety feel less satisfied with their environment and perhaps feel unsafe, are experiencing financial/employment difficulties and are lacking opportunities for recreational activities, all of which were heavily impacted during the COVID-19-related restrictions and lockdowns in Australia (Griffiths et al., 2022). Environmental quality of life also has overlap with psychological and physical quality of life, which may also explain the results.

Having a higher health risk for COVID-19 was found to be associated with poorer quality of life, but only physical quality of life. These findings suggest that those who perceive themselves to have a greater risk of contracting or being negatively affected by COVID-19, reported lower physical quality of life. This is consistent with previous research surrounding COVID-19 in Australia and reflects the degree of physical burden that COVID-19 infection can carry (Auerbach & Miller, 2020). Similarly, it aligns with research suggesting individuals who perceive a greater susceptibility and severity to COVID-19 report lower quality of life outcomes (Kharshiing et al., 2021). In addition, having greater health impairment was also significantly associated with both poorer physical and psychological quality of life. This finding is not surprising and is in-line with previous research which found individuals experiencing chronic disease reported higher levels of distress (Louvardi et al., 2020) and poor physical functioning (Dennison et al., 2022) during COVID-19.

Contrary to our hypotheses, having previous plans to travel (which were subsequently cancelled or postponed) during the pandemic was not significantly associated with, or a predictor of, quality of life during this time. This is inconsistent with recent qualitative and observational studies which suggested the closing of national borders and international travel restrictions negatively influenced quality of life (Klinger et al., 2021). Our findings may be due to the possibility that travel restrictions were only one of the many restrictions and negative outcomes during this time, and thus may have been considered only a minor inconvenience (Fink et al., 2022) compared to other outcomes of COVID-19. When considered in the context of other negative outcomes from COVID-19 (e.g., hospitalisation and physical and personal safety), the health and wellbeing of individuals and their loved ones may be perceived to be more important to them personally, therefore having a greater influence on quality of life (Mohsen et al., 2022). Furthermore, with the introduction and increase of online options during this time, the need for physical travel and attendance at events was substituted with virtual attendance at important events such as weddings, funerals, and graduations (de Haas et al., 2020; Mouratidis, 2021). Perhaps this virtual substitute enabled some of the negative effects of travel restrictions to be reduced, however, further research is required to support this.

## Practical implications and recommendations

The findings from this study provide important insight into numerous practical implications that may be beneficial for maintaining quality of life during times of uncertainty. Given the high importance of adaptive coping, it is important from the outset of a world event that governments, community leaders, media organisations, and practitioners provide the public with ways of coping during such times. Allowing access to free, or subsidised, coping skills classes is one way of doing this (Behzadnia & FatahModares, 2020). Encouraging the public to look for the positive aspects in a negative situation may also be beneficial, for example how spending time with those who are important to you can create stronger relationships for the future. Research further exploring how individuals globally coped with this uncertainty is also important and may inform future clinical practice in similar situations of uncertainty (e.g., war, other epidemics/pandemics). Given the lack of findings for the importance of compensatory behaviours, recommending individuals engage in ‘new’ or ‘substitute’ behaviours to cope is not recommended if improving quality of life is the outcome.

Given the importance of COVID-19 anxiety on many aspects of quality of life, it is recommended that global media organisations aim to reduce the anxiety around uncertain situations by being sensible in their reporting. Whilst it is important to report on current affairs and keep the public informed, research shows that amplified media exposure and reporting during a crisis can lead to increased anxiety and stress in many people (Garfin et al., 2020; Taha et al., 2014; Thompson et al., 2017; Zhao & Zhou, 2020). Providing practical advice to reduce anxiety during such times, such as hand washing during a pandemic or donating to charities during a natural disaster or war, is more beneficial than perpetuating unnecessary levels of fear (Garfin et al., 2020), and subsequently may have more positive effects on quality-of-life outcomes. Policy makers and leaders should insist that media organisations reduce their fear mongering, and instead incorporate practical advice into future media reporting of such events.

Specifically for individuals experiencing a greater perceived risk to their health, practical advice should be health based and not based on fear. Government recommendations for individuals at a heightened physical health risk should be clear, evidence-based, and positive, to ensure the physical quality of life of these individuals is not affected. Similarly, ensuring the continuity of providing and encouraging safer or online alternatives for behaviours that could risk the health and wellbeing of these individuals, such as online therapy, online shopping, exercise in the home, in important moving forward. Health practitioners, such as health psychologists and clinical psychologists, should ensure they do not perpetuate anxiety and stress in those most at risk of COVID-19, but instead help clients work through their fear by focussing on the positives of engaging in particular behaviours.

## Strengths and limitations

This research has several strengths, such as being one of the first studies to explore associations between travel restrictions and quality of life. Secondly, this study assessed multiple domains of quality of life rather than only health related quality of life which has been the focus of many research studies and calls for action during the pandemic (see Amdal et al., 2021; Bryson, 2021). This fills an important gap in the literature by providing information to organisations on how times of uncertainty, such as pandemics, influence different areas of quality of life.

A potential limitation of this study is the cross-sectional nature of the data, limiting the conclusions we can make regarding the influence of travel restrictions on quality of life. In future times of uncertainty, researchers should consider prospective or longitudinal study designs to assess the effects of travel restrictions on quality of life over time. Secondly, the lack of measurement of the degree of importance that individuals place on domestic and international travel is a limitation of this study. Individuals that place more value on travel may have experienced poorer quality of life due to the restrictions. As such, future research should consider exploring this variable in future research related to travel restrictions. Similarly, we did not measure individuals’ tolerance of uncertainty, which may have provided more insight into the psychological factors that influence quality of life during these times. In future similar situations, researchers should consider assessing tolerance of uncertainty to see if individuals with greater tolerance report greater quality of life. Finally, COVID-19 anxiety was the only measure of psychological-based feelings during this time. Perhaps other measures of mental health during this time, such as stress or depression, may have provided greater insight into the relationship between travel restrictions, mental health, and quality of life.

## Conclusion

In this study, we aimed to examine the relationships between COVID-19-related travel restrictions, psychological variables and quality of life in Australian adults. The proposed model showed that better adaptive coping, decreased COVID-19 anxiety, fewer health impairments, and lower perceived health risk for COVID-19 were all associated with better quality of life. Surprisingly, travel plans did not explain a significant proportion of variance in quality of life. This may be due to a shift in priorities that many people have experienced throughout the COVID-19 pandemic (e.g., a greater focus on the health and wellbeing of themselves). The findings provide directions and recommendations for strategies that governments and media organisations can use to increase adaptative coping and decrease anxiety and health risk when future events, such as wars or natural disasters, occur.

## References

[CR1] Amdal CD, Pe M, Falk RS, Piccinin C, Bottomley A, Arraras JI, Darlington AS, Hofsø K, Holzner B, Jørgensen NMH, Kulis D, Rimehaug SA, Singer S, Taylor K, Wheelwright S, Bjordal K (2021). Health-related quality of life issues, including symptoms, in patients with active COVID-19 or post COVID-19; a systematic literature review. Quality of Life Research.

[CR2] Auerbach J, Miller BF (2020). COVID-19 Exposes the Cracks in Our Already Fragile Mental Health System. American Journal of Public Health.

[CR3] Australian Bureau of Statistics (2021). *Overseas Arrivals and Departures, Australia*.

[CR4] Australian Institute of Health and Welfare (2020). *People with disability in Australia 2020: in brief*.

[CR5] Behzadnia B, FatahModares S (2020). Basic Psychological Need-Satisfying Activities during the COVID‐19 Outbreak. Applied Psychology: Health and Well-Being.

[CR6] Bou-Karroum L, Khabsa J, Jabbour M, Hilal N, Haidar Z, Abi Khalil P, Khalek RA, Assaf J, Honein-AbouHaidar G, Samra CA, Hneiny L, Al-Awlaqi S, Hanefeld J, El-Jardali F, Akl EA, el Bcheraoui C (2021). Public health effects of travel-related policies on the COVID-19 pandemic: A mixed-methods systematic review. Journal of Infection.

[CR7] Brosschot JF, Verkuil B, Thayer JF (2016). The default response to uncertainty and the importance of perceived safety in anxiety and stress: An evolution-theoretical perspective. Journal of Anxiety Disorders.

[CR8] Bryson WJ (2021). Long-term health-related quality of life concerns related to the COVID-19 pandemic: a call to action. Quality of Life Research.

[CR9] Calear AL, McCallum S, Morse AR, Banfield M, Gulliver A, Cherbuin N, Farrer LM, Murray K, Harris R, Batterham PJ (2022). Psychosocial impacts of home-schooling on parents and caregivers during the COVID-19 pandemic. Bmc Public Health.

[CR10] Cho H, Oh GE, Grace, Chiu W (2021). Compensatory consumption during the COVID-19 pandemic: exploring the critical role of nostalgia in sport consumer behaviour. Journal of Marketing Management.

[CR11] Chua BL, Al-Ansi A, Lee MJ, Han H (2021). Impact of health risk perception on avoidance of international travel in the wake of a pandemic. Current Issues in Tourism.

[CR12] Coghlan A (2015). Tourism and health: using positive psychology principles to maximise participants’ wellbeing outcomes – a design concept for charity challenge tourism. Journal of Sustainable Tourism.

[CR13] Cohen, J. (1988). *Statistical power for the behavioural sciences*. Lawrence Erlbaum Associates Publishers.

[CR14] de Haas M, Faber R, Hamersma M (2020). How COVID-19 and the Dutch ‘intelligent lockdown’ change activities, work and travel behaviour: Evidence from longitudinal data in the Netherlands. Transportation Research Interdisciplinary Perspectives.

[CR15] Dennison I, Schweizer C, Fitz T, Blasko D, Sörgel C, Kallies A, Schmidt L, Fietkau R, Distel LV (2022). Substantial Impairment of Quality of Life during COVID-19 Pandemic in Patients with Advanced Rectal Cancer. Healthcare.

[CR16] Faasse, K., & Newby, J. (2020). Public Perceptions of COVID-19 in Australia: Perceived Risk, Knowledge, Health-Protective Behaviors, and Vaccine Intentions. *Frontiers in Psychology*, *11*. 10.3389/fpsyg.2020.551004 10.3389/fpsyg.2020.551004 PMC7561403 33117223

[CR17] Fink G, Tediosi F, Felder S (2022). Burden of Covid-19 restrictions: National, regional and global estimates. EClinicalMedicine.

[CR18] Fischer ME, Cruickshanks KJ, Klein BEK, Klein R, Schubert CR, Wiley TL (2009). Multiple sensory impairment and quality of life. Ophthalmic Epidemiology.

[CR19] Fisher JR, Tran TD, Hammarberg K, Sastry J, Nguyen H, Rowe H, Popplestone S, Stocker R, Stubber C, Kirkman M (2020). Mental health of people in Australia in the first month of < scp > COVID -19 restrictions: a national survey. Medical Journal of Australia.

[CR20] Fredrickson BL (2001). The role of positive emotions in positive psychology: The broaden-and-build theory of positive emotions. American Psychologist.

[CR21] Garfin DR, Silver RC, Holman EA (2020). The novel coronavirus (COVID-2019) outbreak: Amplification of public health consequences by media exposure. Health Psychology.

[CR22] Griffiths D, Sheehan L, Petrie D, van Vreden C, Whiteford P, Collie A (2022). The health impacts of a 4-month long community-wide COVID-19 lockdown: Findings from a prospective longitudinal study in the state of Victoria, Australia. PLOS ONE.

[CR23] Hagedorn RL, Wattick RA, Olfert MD (2022). “My Entire World Stopped”: College Students’ Psychosocial and Academic Frustrations during the COVID-19 Pandemic. Applied Research in Quality of Life.

[CR24] Hammad J, Tribe R (2021). Adaptive coping during protracted political conflict, war and military blockade in Gaza. International Review of Psychiatry.

[CR25] Ioannides D, Gyimóthy S (2020). The COVID-19 crisis as an opportunity for escaping the unsustainable global tourism path. Tourism Geographies.

[CR26] Javed S, Parveen H (2021). Adaptive coping strategies used by people during coronavirus. Journal of Education and Health Promotion.

[CR27] Kharshiing KD, Kashyap D, Gupta K, Khursheed M, Shahnawaz MG, Khan NH, Uniyal R, Rehman U (2021). Quality of Life in the COVID-19 Pandemic in India: Exploring the Role of Individual and Group Variables. Community Mental Health Journal.

[CR28] Klinger, C., Burns, J., Movsisyan, A., Biallas, R., Norris, S. L., Rabe, J. E., Stratil, J. M., Voss, S., Wabnitz, K., Rehfuess, E. A., & Verboom, B. (2021). Unintended health and societal consequences of international travel measures during the COVID-19 pandemic: a scoping review. *Journal of Travel Medicine*, *28*(7), 10.1093/jtm/taab123 10.1093/jtm/taab123 PMC8436381 34369562

[CR29] Kowal M, Coll-Martín T, Ikizer G, Rasmussen J, Eichel K, Studzińska A, Koszałkowska K, Karwowski M, Najmussaqib A, Pankowski D, Lieberoth A, Ahmed O (2020). Who is the Most Stressed During the COVID‐19 Pandemic? Data From 26 Countries and Areas. Applied Psychology: Health and Well-Being.

[CR30] Kwok WC, Wong CK, Ma TF, Ho KW, Fan LWT, Chan KPF, Chan SSK, Tam TCC, Ho PL (2021). Modelling the impact of travel restrictions on COVID-19 cases in Hong Kong in early 2020. Bmc Public Health.

[CR31] Lazarus, R., & Folkman, S. (1984). *Stress, Appraisal, and Coping*. Springer Publishing Company.

[CR32] Lenggogeni S, Ashton AS, Scott N (2022). Humour: coping with travel bans during the COVID-19 pandemic. International Journal of Culture Tourism and Hospitality Research.

[CR33] Lew AA (2018). Why travel? – travel, tourism, and global consciousness. Tourism Geographies.

[CR34] Louvardi M, Pelekasis P, Chrousos GP, Darviri C (2020). Mental health in chronic disease patients during the COVID-19 quarantine in Greece. Palliative and Supportive Care.

[CR35] Macía P, Barranco M, Gorbeña S, Iraurgi I (2020). Expression of resilience, coping and quality of life in people with cancer. PLOS ONE.

[CR36] Marques L, Bartuska AD, Cohen JN, Youn SJ (2020). Three steps to flatten the mental health need curve amid the COVID-19 pandemic. Depression and Anxiety.

[CR37] Meyer D, van Rheenen TE, Neill E, Phillipou A, Tan EJ, Toh WL, Sumner PJ, Rossell SL (2022). Surviving the COVID-19 pandemic: An examination of adaptive coping strategies. Heliyon.

[CR38] Mohamadzadeh Tabrizi Z, Mohammadzadeh F, Davarinia Motlagh Quchan A, Bahri N (2022). COVID-19 anxiety and quality of life among Iranian nurses. BMC Nursing.

[CR39] Mohsen S, El-Masry R, Ali OF, Abdel-Hady D (2022). Quality of life during COVID-19 pandemic: a community-based study in Dakahlia governorate, Egypt. Global Health Research and Policy.

[CR40] Mouratidis K (2021). How COVID-19 reshaped quality of life in cities: A synthesis and implications for urban planning. Land Use Policy.

[CR41] Osofsky JD, Osofsky HJ, Mamon LY (2020). Psychological and social impact of COVID-19. Psychological Trauma: Theory Research Practice and Policy.

[CR42] Özdin S, Bayrak Özdin Ş (2020). Levels and predictors of anxiety, depression and health anxiety during COVID-19 pandemic in Turkish society: The importance of gender. International Journal of Social Psychiatry.

[CR43] Peteet JR (2020). COVID-19 Anxiety. Journal of Religion and Health.

[CR44] Piret, J., & Boivin, G. (2021). Pandemics Throughout History. *Frontiers in Microbiology*, *11*. 10.3389/fmicb.2020.631736 10.3389/fmicb.2020.631736 PMC7874133 33584597

[CR45] Sahni PS, Singh K, Sharma N, Garg R (2021). Yoga an effective strategy for self-management of stress-related problems and wellbeing during COVID19 lockdown: A cross-sectional study. PLOS ONE.

[CR46] Same A, McBride H, Liddelow C, Mullan B, Harris C (2020). Motivations for volunteering time with older adults: A qualitative study. PLOS ONE.

[CR47] Shamblaw AL, Rumas RL, Best MW (2021). Coping during the COVID-19 pandemic: Relations with mental health and quality of life. Canadian Psychology/Psychologie Canadienne.

[CR48] Shek DTL (2021). COVID-19 and Quality of Life: Twelve Reflections. Applied Research in Quality of Life.

[CR49] Shoychet G, Lenton-Brym AP, Antony MM (2022). The impact of COVID-19 anxiety on quality of life in Canadian adults: The moderating role of intolerance of uncertainty and health locus of control. Canadian Journal of Behavioural Science / Revue Canadienne Des Sciences Du Comportement.

[CR50] Silva WAD, de Sampaio Brito TR, Pereira CR (2020). COVID-19 anxiety scale (CAS): Development and psychometric properties. Current Psychology.

[CR51] Sinclair VG, Wallston KA (2004). The Development and Psychometric Evaluation of the Brief Resilient Coping Scale. Assessment.

[CR52] Taha SA, Matheson K, Anisman H (2014). H1N1 Was Not All That Scary: Uncertainty and Stressor Appraisals Predict Anxiety Related to a Coming Viral Threat. Stress and Health.

[CR53] THE WHOQOL GROUP (1998). Development of the World Health Organization WHOQOL-BREF Quality of Life Assessment. Psychological Medicine.

[CR54] Thompson RR, Garfin DR, Holman EA, Silver RC (2017). Distress, Worry, and Functioning Following a Global Health Crisis: A National Study of Americans’ Responses to Ebola. Clinical Psychological Science.

[CR55] Tintori A, Cerbara L, Ciancimino G, Cresimbene M, la Longa F, Versari A (2020). Adaptive behavioural coping strategies as reaction to COVID-19 social distancing in Italy. European Review for Medical and Pharmacological Sciences.

[CR56] Vieira DA, Meirinhos V (2021). COVID-19 Lockdown in Portugal: Challenges, Strategies and Effects on Mental Health. Trends in Psychology.

[CR57] Zhao N, Zhou G (2020). Social Media Use and Mental Health during the COVID-19 Pandemic: Moderator Role of Disaster Stressor and Mediator Role of Negative Affect. Applied Psychology: Health and Well-Being.

